# Canine Plasminogen: Spectral Responses to Changes in 6-Aminohexanoate and Temperature

**Published:** 2007-03-22

**Authors:** Jack A. Kornblatt, Tanya A. Barretto, Ketevan Chigogidze, Bahati Chirwa

**Affiliations:** Enzyme Research Group, Department of Biology, Concordia University, Montreal, Qc., Canada H4B 1R6.

**Keywords:** plasminogen, conformational change, fourth derivative spectroscopy, temperature effects

## Abstract

We studied the near UV absorption spectrum of canine plasminogen. There are 19 tryptophans, 19 phenylalanines and 34 tyrosines in the protein. 4th derivative spectra optimized for either tryptophan or tyrosine give a measure of the polarity of the environments of these two aromatic amino acids. Plasminogen at temperatures between 0 °C and 37 °C exists as a mixture of four conformations: closed-relaxed, open-relaxed, closed-compact, and open-compact. The closed to open transition is driven by addition of ligand to a site on the protein. The relaxed to compact transition is driven by increasing temperature from 0 °C to above 15–20 °C.

When the conformation of plasminogen is mainly closed-relaxed, the 4th derivative spectra suggest that the average tryptophan environment is similar to a solution of 20% methanol at the same temperature. Under the same conditions, 4th derivative spectra suggest that the average tyrosine environment is similar to water. These apparent polarities change as the plasminogen is forced to assume the other conformations. We try to rationalize the information based on the known portions of the plasminogen structure.

## Introduction

Plasminogen is a blood protein; it is the precursor of the proteolytic enzyme plasmin (extensively reviewed by (Bachmann, 1994; Castellino and McCance, 1997; Castellino and Powell, 1981; Markus et al. 1978; Markus, 1996). Plasmin fills multiple roles. A major function is to clear the *in vivo* milieu of unwanted fibrin clots. Should these occur in the lungs, brain or heart, they can give rise to lethal sequelae. Plasmin can also degrade the tight junctions between cells and thereby facilitate tissue remodeling (Bass and Ellis, 2002). During growth and development, this is a positive function (Estelles et al. 1980; Nakagami et al. 2000). During tumour metastasis it is a negative function. During infection by staphylococci or streptococci, it may be a mixed blessing (Bergmann et al. 2004). During prion propagation the presence of plasminogen accelerates the rate at which the scrapie form gets to the brain but does not influence the final outcome of the disease (Salmona et al. 2005). Plasminogen binds to many proteins: enolase (Andronicos et al. 1997; Miles et al. 1991; Redlitz et al. 1995), streptokinase and staphylokinase (Boxrud et al. 2000), cellular prions (Ellis et al. 2002; Fischer et al. 2000; Kornblatt et al. 2003; Maissen et al. 2001; Praus et al. 2003; Ryou et al. 2003) as well as a host of others containing either exposed lysyl groups or amino sugars. Whether this binding fulfills a functional role is not clear in many cases. Certainly, when plasminogen binds to fibrin clots, it can easily be activated to plasmin.

There are several routes to activation of plasminogen to plasmin. Activation involves either proteolytic attack at the bond between residues 561 and 562 of the intact protein or binding of plasminogen to streptokinase without cleavage (Boxrud et al. 2000). If the “native” form of glu-plasminogen has first been converted to lys-plasminogen, the activation is faster than if the bond between residues 77 and 78 has not been cleaved (Gong et al. 2001; Miles et al. 2003). In any event, opening of the protein from its compact, closed form is probably obligatory to exposing the 561–562 bond.

Glu-PGN is the 791 amino acid protein that is initially secreted into blood. It is organized into several domains (Bachmann, 1994): The N-terminal peptide (residues 1–83) is followed sequentially by five tightly packed kringle domains; a linker region connects kringle 5 to the proteolytic domain. The terminology is interesting: A kringle domain is defined as a stretch of 80–90 amino acids organized into two pairs of antiparallel β-strands; the domain contains three disulfide bridges which, in a two dimensional representation, give the kringle its Danish name meaning pretzel. Lys-PGN lacks the first 77–78 N-terminal amino acids.

**Scheme 1:**
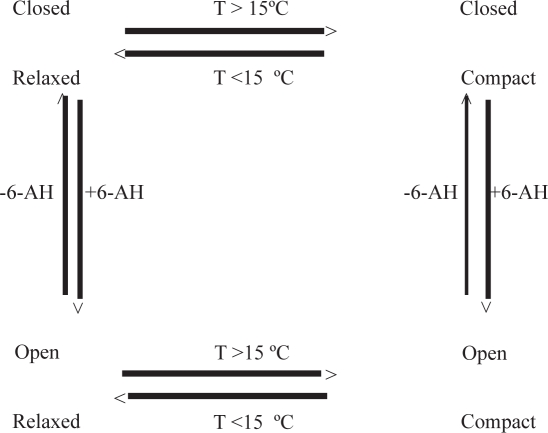


Just as plasminogen has many proteins to which it can bind, it has several well defined and less well defined conformations. Glu-plasminogen can be either open or closed (Alkjaersig et al. 1959) (Scheme 1). When closed, lys-50 occupies a binding site on the PGN domain kringle 5 (An et al. 1998; Cockell et al. 1998) and the entire plasminogen assumes a lock washer like form (Tranqui et al. 1979b; Weisel et al. 1994). When the kringle 5 binding site is occupied by either another lysine, lysine analog, amino sugar or water, the molecule opens and assumes a horse shoe shape (Tranqui et al. 1979a; Tranqui et al. 1979b; Weisel et al. 1994). Lys-PGN, since it lacks lysine 50, is always open. The open-closed transition was discovered in 1959 by Alkjaersig (Alkjaersig et al. 1959). The three principle conformations have been extensively studied (Brockway and Castellino. 1971; Castellino et al. 1973; Christensen and Molgaard, 1991; Markus et al. 1979; Markus, 1996; Molgaard et al. 1993; Violand et al. 1978). Plasminogen can also exist in three other less well studied conformations (Kornblatt and Schuck, 2005; Marshall et al. 1994). One has been defined on the basis of its structure in the presence of benzamidine, the other two are found at low temperature and are relaxed conformational variants of the classical open and closed forms. This work will be concerned with only the four conformations related by temperature and 6-AH and will not cover lys-plasminogen nor the variant resulting from the binding of benzamidine. The conformational transitions relevant to this work are shown in Scheme 1.

We started to study DPGN because we thought that it might be easier to crystallize than holo HPGN. There are crystal structures or NMR solution structures for all the kringles and the catalytic domain (Abad et al. 2002; Chang et al. 1998; Marti et al. 1999; Mathews et al. 1996; Stec et al. 1997; Wang and Reich, 1995) but the complete protein has not been solved. Clearly, with at least four interconnected conformations in which the energy barriers between the conformations are small, it will require both ingenuity and insight to arrive at a structure for the intact molecule.

In order to predict the response of plasminogen to perturbation and to find conditions that might reduce the number of conformations in any given mix, we have looked at the response of the aromatic amino acids to temperature change and the ligand 6-AH. For this purpose, we have used 4th derivative, near UV spectroscopy (Butler, 1979). This technique has been used extensively in order to determine the environments of aromatic amino acid residues and how these change during folding/unfolding and in response to perturbants such as hydrostatic pressure (Chatani et al. 2002; Fujiwara et al. 2001; Herberhold et al. 2003; Marchal et al. 2003; Mombelli et al. 1997; Torrent et al. 1999). 4th derivative absorption spectroscopy has also found use in determining the response of proteins to conformational change (Casati et al. 2000; Derrick et al. 2004; Di Pietro and Santome, 2000; Dunach et al. 1983; Mombelli et al. 1997; Padros et al. 1982; Ruan et al. 2002; Torrent et al. 2005). Lastly it has been used to study the effects of mutations on the overall environments of the aromatics (Kornblatt et al. 1999). Our data indicate that binding of 6-AH to DPGN results in significant shifts in the environments of the aromatic residues of this molecule.

## Materials and Methods

Canine plasminogen was purified from dog blood as described (Castellino and Powell, 1981). Aprotinin (Sigma Chem. Co.) was added to a final concentration of 0.3 μM. The protein, after purification, was freeze dried and stored in flame sealed vials under vacuum. It was dialyzed 3 × vs 5 mM K_2_HPi, 5 mM KH_2_Pi, 300 mM NaCl before use. SDS-PAGE was carried out on the purified protein after each experiment. If there was any indication of conversion to lys-plasminogen or plasmin, the data were discarded.

Absorption spectra, on mixtures of (N-acetyl-O-ethyl tryptophan/N-acetyl-O-ethyl tyrosine/N-acetyl-O-ethyl phenylalanine) or on DPGN, were obtained from 315 nm to 255 nm on a Cary 2400 UV/VIS/NIR spectrophotometer. Data were collected at 0.1 nm intervals with a response time of 1 s, a slit of 1 nm, and a collection rate of 0.1 nm/s. The spectrophotometer was thermostated with a Lauda RK20 circulating bath. The temperature was measured with an in situ thermocouple in the sample compartment. The zero order spectra were converted to 4th derivative spectra using a numerical treatment of the data described by Lange (Lange et al. 1996). In essence the procedure consists of taking the zero order spectrum, duplicating it and displacing it along the wavelength axis by a given number of nanometers. The displacement is 2.6 nm when we wish to emphasize changes in the spectrum that are the result of changes in the environment of tyrosine. The displacement is 1.6 nm when the emphasis is tryptophan. The displaced zero order spectrum is then subtracted from the original thereby yielding a first derivative spectrum. The process is repeated treating the new derivative spectrum as the original, duplicating and displacing it and then subtracting once again to yield a second derivative spectrum. The process is repeated until the fourth derivative spectrum is obtained. The treatment of the data has been optimized such that one can emphasize the changes that occur in average tyrosine or tryptophan spectra. This in turn reflects changes in the average environment of the tyrosine or tryptophan. The derivatives are highly accurate provided the data intervals of the zero order spectra are sufficiently closely spaced. For most purposes, a data interval of 0.1 nm per point is adequate. To give some feel for the precision of the resultant spectra, if we take 10 absorption spectra of the same sample at the same temperature on the same day, we cannot distinguish by eye any difference in the derivative spectra. A plot of the peak ratios (vida infra) emphasizing either tryptophan or tyrosine yields data in which the spread is less than 2%. The aromatic amino acid mixture contained the N-acetyl-O-ethyl-esters of tryptophan, tyrosine and phenylalanine in a ratio of (19:34:19) which is the same as occurs in dog plasminogen. We checked that the mixture was precise by comparing the spectral sum of the individual components with that of the mixture. In this case, the sum of the parts was equal to the whole. The 4th derivative of the mix differed by less than 1% from the sum of the individual components. The original paper (Lange et al. 1996) should be consulted for details on both the precision and power of the approach.

**Figure 1.**
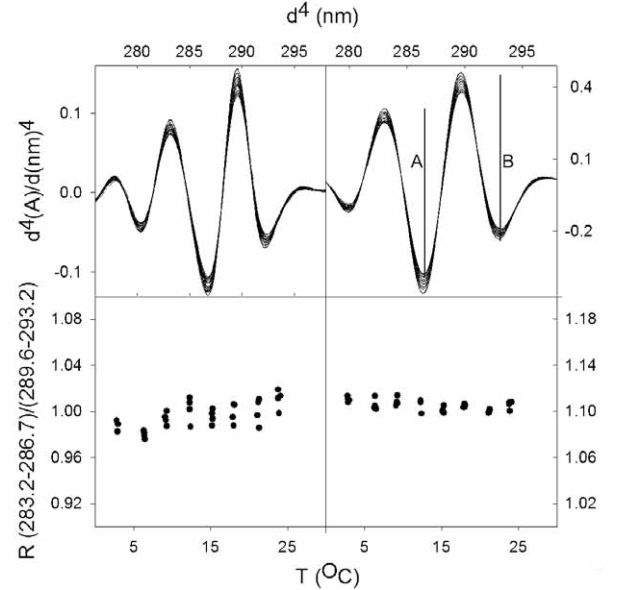
Fourth derivative absorption spectra and R-values of a mixture of tryptophan, tyrosine and phenylalanine (19:34:19) in 50% Methanol, 50% water as a function of temperature. The absorbances at the two major peaks and two major troughs in the upper panels are the values used to calculate the R-values shown in the lower panels. The zero order spectra on which the derivatives are based are not shown. The panels on the left side were optimized such that they emphasize tryptophan spectra. The panels on the right side emphasize tyrosine spectra. The two drop lines in the spectra indicate the peak to trough values that form the basis of the “R = A/B” calculation.

The 4th derivative data were evaluated on the basis of a parameter “R”. It is a ratio of selected peak to trough values of the 4th derivative spectra. In Figure 1:

$$R\;=\;A/B\;=\;(A_{283.2nm}-A_{286.7nm})/(A_{289.6nm}-A_{293.2nm})$$

where the ratio, A/B refers to the algebraic peak to trough differences shown by the drop lines in the figure. The exact peak and trough positions vary with environment but are well documented indicators of polarity (Kornblatt et al. 1999; Lange et al. 1996; Schroeder et al. 1997).

## Results

We wished to determine how the environments of the aromatic amino acids changed as DPGN went from a high temperature, closed and compact structure to a low temperature, closed and relaxed structure. Further, what changes in environment occurred as the closed structures opened? The strategy used to answer these questions was as follows: We measured the spectra of closed and open DPGN at different temperatures; we then compared the spectra to those of synthetic mixtures of aromatic amino acids at different temperatures and different concentrations of methanol. Temperature influences many characteristics of our solutions (Douzou, 1977) as does methanol. 6-AH was used to bring about the closed to open transition; it influences the dielectric coefficient of our solutions; adding 50 mM 6-AH causes the solution’s dielectric constant to go from 80.4 to 81.5 at 20 °C and from 88.1 to 89.2 at 0 °C. By contrast, going from water at 20 °C to 50% methanol at 20 °C, the dielectric constant goes from 80.4 to 60.3 (Douzou.1977).

A stock mixture of the N-acetyl-O-ethylesters of 19 mM tryptophan, 34 mM tyrosine and 19 mM phenylalanine in 100% methanol was used for comparative purposes; this mixture mimics the ratio of aromatics in canine plasminogen (Carter and Kornblatt, 2005). For absorption work, the stock was diluted into mixtures of methanol:water such that the A_280 nm_ was close to 1. Stray light error was minimal and measurement precision was optimal at this absorbance. Figure 1 shows a representative set of data taken with a mixture of 50% methanol: 50% water. The zero order spectra are not shown. The two upper panels show the 4th derivative data for several different temperatures; the left side panels emphasize the changes occurring in the tryptophan spectra while the emphasis on tyrosine is shown in the right side panels. The lower panels show the response of the parameter, R, to temperature. R (see Methods) is the ratio of peak to trough absorbances (Fisher and Sligar, 1985; Ragone et al. 1984) (Schroeder et al. 1997). The two sets of derivatives were obtained from the same set of zero order spectra. They differ in the displacement of the duplicated spectrum (see Methods) used for the derivative (Lange et al. 1996): A displacement of 1.6 nm emphasizes the changes occurring in tryptophan spectra; a displacement of 2.6 nm emphasizes the changes occurring in tyrosine spectra. It goes without saying that this is an approximation. All three aromatics contribute to the spectra and have contributions present in the derivatives.

The feature of the data that is most striking is that the precision of the determinations is quite high and quite reproducible. Within a given data set, the R value rarely deviates from the mean at a single temperature by more than 1%. The second feature that is apparent is that the R value is not constant over the temperature range. As the temperature increases from 0 °C to 24 °C, the R value increases by 4% for tryptophan emphasis and decreases slightly for tyrosine emphasis (Figure 1).

**Figure 2.**
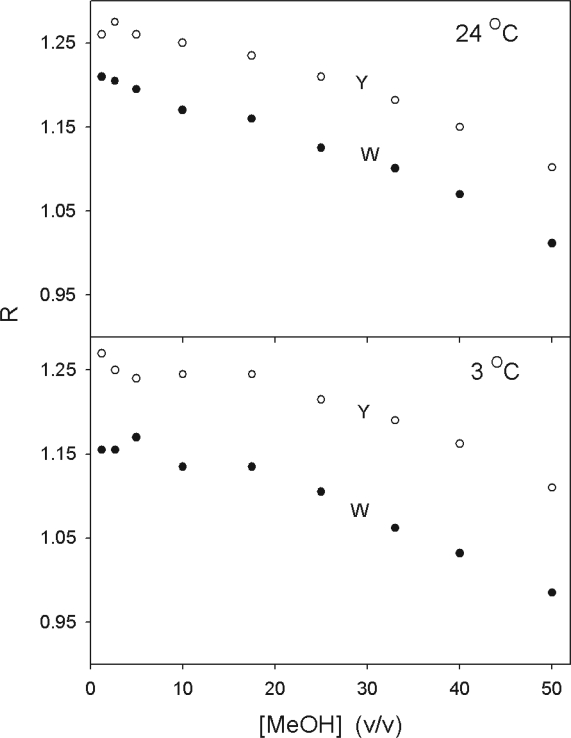
The R-values of both tryptophan-emphasis and tyrosine-emphasis, obtained from derivative absorption spectra, are a function of the solvent polarity and temperature. The upper panel (24 °C) shows the evolution of R for both tryptophan and tyrosine emphasis as a function of the concentration of methanol. The lower panel shows the evolution of R as a function of methanol concentration at 3 °C.

Figure 2 shows R-value data summarized for concentrations of methanol between 0 and 50% methanol. The only data that are shown are for two temperatures, 3 °C and 24 °C. Data between these two temperatures at 2 °C intervals were taken but are not shown. There is a steady decline in R as the methanol concentration is increased. R monitors the polarity of the solvent and the polarity decreases as the concentration of methanol increases.

**Figure 3.**
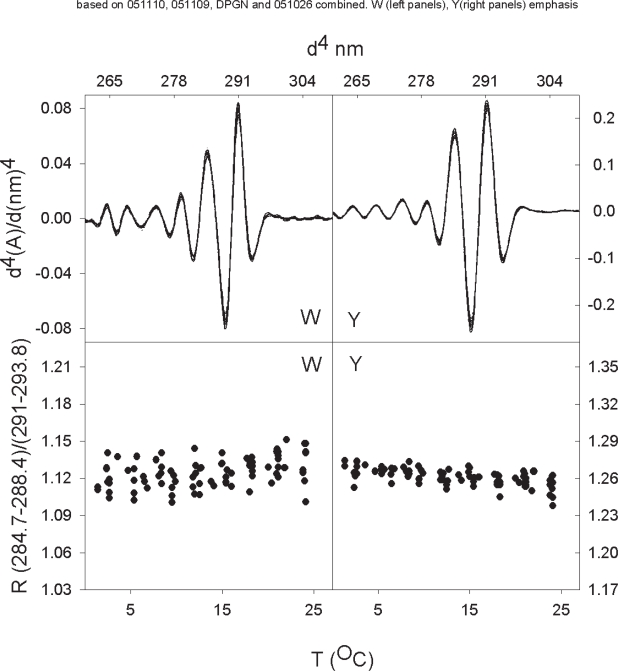
Fourth derivative absorption spectra and R-values of (closed) DPGN as a function of temperature. The absorbances at the two major peaks and two major troughs in the upper panels are the values used to calculate the R-values shown in the lower panels. The zero order spectra on which the derivatives are based are not shown. The panels on the left side were optimized such that they emphasize tryptophan spectra. The panels on the right side emphasize tyrosine spectra.

In Figure 3 we present the 4th derivatives and R values of closed DPGN as a function of temperature. The conformation of DPGN is closed-relaxed at the low temperature and closed-compact at the higher temperature. There are two significant features of the R-value data: The tryptophan emphasis data in the lower left panel indicate that the R-value is about 1.12 at 3 °C and that this value is more or less constant up to 24 °C. This value is consistent with an environment of 20% methanol at the lower temperature and about 25% methanol at the higher temperature; a 5% change in our comparative value is not significant. The second feature of the data that is noteworthy is that the tyrosine R-values correspond to tyrosine in an aqueous milieu and this does not change as a function of temperature. The average environments for the two amino acids are summarized in Table 1.

**Figure 4.**
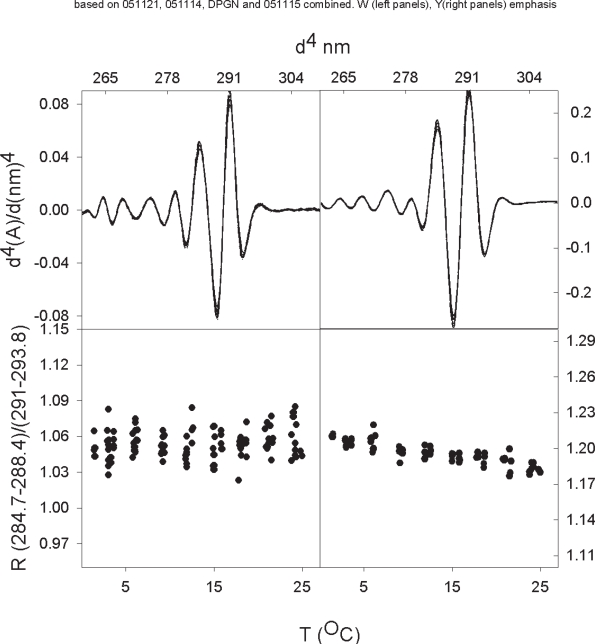
Fourth derivative absorption spectra and R-values of (open) DPGN as a function of temperature. The samples contained 50 mM 6-aminohexanoate which is sufficient to saturate the four kringle binding sites. The absorbances at the two major peaks and two major troughs in the upper panels are the values used to calculate the R-values shown in the lower panels. The zero order spectra on which the derivatives are based are not shown. The panels on the left side were optimized such that they emphasize tryptophan spectra. The panels on the right side emphasize tyrosine spectra.

When 6-aminohexanoate is added, such that all four kringles are saturated, there is a significant shift in the R-values (Figure 4 and Table 1). The tryptophan environment appears to go from about 20–25% methanol to 35–40% methanol. The environment of the average tyrosine increases from close to 0% methanol to about 30% methanol indicating a major decrease in polarity. A comparison of Figures 3 and 4 shows quite clearly that there is no overlap in the data at any temperature. Both of these shifts in the comparative values are significant.

**Figure 5.**
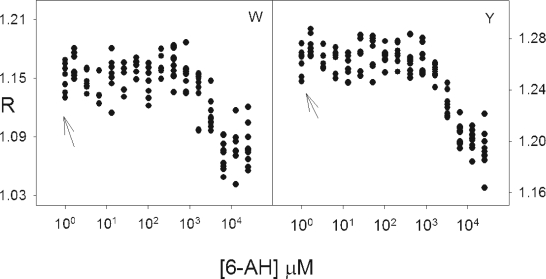
The R-values of DPGN as a function of 6-AH concentration. Four binding events can be discerned in both panels as the protein goes from the fully closed and compact structure to the fully open and compact form. Inflection points show that the first binding event occurs in the region slightly greater than 10^0^ μM The second occurs before 10^1^ μM. The third occurs in the region 10^2^ μM and the fourth in the region 10^3.5^ μM. The changes occurring in tryptophan are emphasized in the left panel, the changes in tyrosine in the right panel.

We tried to resolve which aromatic residues contributed most heavily to the changes in R seen in the closed to open transition. The rationale was based on the fact that the different kringles bind 6-AH with different affinities and that the protein does not assume the open conformation until kringle 5, which has the lowest affinity, binds the ligand (Kornblatt, 2000). The order of binding is kringle 4 first, kringle 1 second, kringle 2 third and kringle 5 last; kringle 3 does not bind 6-AH to any significant extent. DPGN was titrated with 6-AH and the absorption spectra determined as a function of [6-AH]. The evolution of the 4th derivative, expressed as R, as a function of concentration is shown in Figure 5. The arrows in the figure indicate the R values of the samples with zero 6-AH. The R values for the right hand panel, emphasizing tyrosine, are considerably tighter than those for tryptophan but, it should be recalled, both R value data sets originate from the same zero order spectra.

In Figure 5, the average values of R show increases as [6-AH] increases from 0 μM to 1.6 μM 6-AH before they begin to decrease. This corresponds to changes occurring in the binding pocket of kringle 4 whose dissociation constant for 6-AH is 3 μM to 7 μM (Kornblatt, 2000; Kornblatt et al. 2001a). As binding occurs, the pocket appears to become more polar (Figure 5). Kringle 4 has two tryptophans as part of the binding pocket and one located just behind it (Wu et al. 1991). It also has two tyrosines located close by with three others further away. The increased polarity of the binding pocket is sensed by both tryptophans and tyrosines. This is reflected in an increase in fluorescence shown by this kringle when the intact protein binds 6-AH (Kornblatt et al. 2001a); the increased fluorescence has been rationalized on the basis of changes in hydrogen bonding patterns when the kringle binds 6-AH (Kornblatt, 2000).

Figure 5 shows that there is a second change in R that culminates at about 15 μM–20 μM. This corresponds to the changes occurring in kringle 1 whose Kd for 6-AH is about 15 μM. The decrease in R at 15 μM corresponds to a drop in polarity (Figure 5). The binding pocket of kringle 1 contains one tryptophan and one tyrosine; there are an additional two tyrosines at the pocket sides as well as one additional tryptophan and another two tyrosines located somewhat farther away (Mathews et al. 1996). The polarity of the pocket is reduced (Figure 5) but the fluorescence of this kringle decreases when the intact protein binds 6-AH at kringle 1(Kornblatt et al. 2001b). Once again, this change in fluorescence has been explained on the basis of changing hydrogen bonding patterns (Kornblatt, 2000).

The third binding event occurs with a Kd of 0.85 mM and shows an increase in R values for both tryptophan and tyrosine. Kringle 2 contains three tryptophans and three tyrosines; it shows the same increase in R for both aromatics as does kringle 4. The last binding event occurs at kringle 5 and has a dissociation coefficient of about 4 mM. It resembles kringle 1 in structure in that the binding pocket consists of one tyrosine and one tryptophan as well as two tyrosines at the sides of the pocket. There is a large decrease in R when the protein binds 6-AH at kringle 5 even though this is an exchange reaction in which lysine 50 is exchanged out for 6-AH.

## Discussion

In this study, we followed the spectral characteristics of plasminogen as a function of temperature and the closed/open transition. We chose to look at temperature effects because plasminogen undergoes a striking relaxed to compact transition as temperature is increased. The Stokes’ radius of the protein decreases by about 25% as the temperature goes from 2 °C to 37 °C. We chose to look at the closed to open transition because, here too, the molecule undergoes a large conformational change. In all, there are four interconverting conformers in equilibrium at 2 °C to 37 °C.

**Figure 6.**
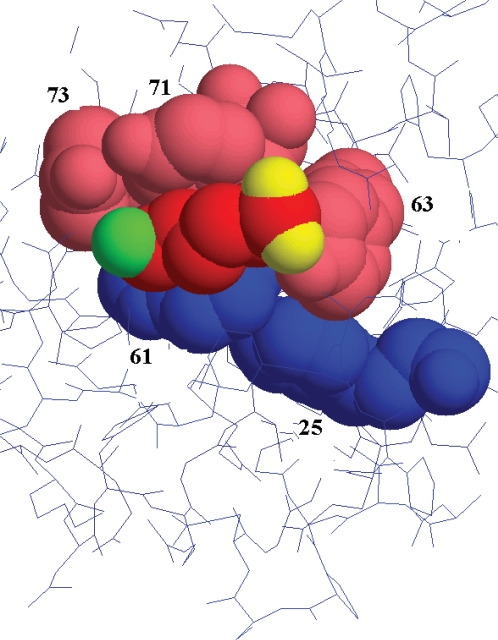
The binding crevice of kringle 1 showing the disposition of 6-AH. The structure is based on the data of Mathews et al. (Mathews et al. 1996). The methylene carbons of 6-AH are red, the ε-amino group is green and the carboxylate oxygens are yellow. Tryptophans are colored blue and tyrosines are pink. 6-AH is sandwiched between tryptophan 61 and tyrosine 71. Tyrosines 63 and 73 are located at the carboxy- and amino- ends of 6-AH while tryptophan 25 is located beneath the site. The numbers adjacent to the amino acids indicate the residue number. In the absence of 6-AH, the crevice is exposed to solvent.

We will restrict the discussion to kringles 1, 2, 4, and 5 because neither kringle 3 nor the proteolytic domain, nor the NTP binds 6-AH. The disposition of 6-AH in the binding crevice of kringle 1 is shown in Figure 6; the binding crevices of all the kringles are quite similar such that Figure 6 is an approximation to all the kringles. Of the 19 tryptophans and 34 tyrosines in DPGN, nine tryptophans and 16 tyrosines are in one of the three non-binding domains and these will not dealt with even though they may account for some of the temperature induced spectral changes or indirectly as a result of the protein opening.

We chose to use derivative spectroscopy of the absorption spectra because it reports on the environments of all the aromatic residues in the protein. It is important to emphasize that the information about the environments of the tryptophans and tyrosines derives from the same zero order spectrum. The data are then treated such that the emphasis gives a number that is more representative of the tryptophan environment or the tyrosine environment. The separation into effects on tryptophan or effects on tyrosine must be taken cum grano salis.

## We first review the tryptophan data

The apparent environment of the average tryptophan in closed DPGN does not change significantly when temperature is changed (Figure 3). The apparent environment of the average tryptophan in plasminogen becomes less polar as the protein opens on addition of 6-AH (Figures 3, 4 and 5). 6-AH is a homolog of lysine; each end carries a full charge but the five methylene carbons of 6-AH are apolar. Figure 6 shows the structure of 6-AH as it sits in the binding crevice of kringle 1. The five methylene carbons are cradled by tyrosine 71 and tryptophan 61 of the binding pocket. In the absence of 6-AH, the pocket is exposed to aqueous solvent.

Kringles 1 and 5 contain two tryptophans; kringles 2 and 4 contain three each. The structures of these four kringles are quite similar but not identical (Kornblatt.2000). In kringle 1, one tryptophan and one tyrosine form the binding site for 6-AH; two other tyrosines sit under the carboxy-carbon and the amino-carbon while the second tryptophan lies just under the binding site tryptophan. These features are shown in Figure 6. In the absence of 6-AH, the site is occupied by water. On binding 6-AH the environments of the two tryptophans change substantially as water at the site is displaced by 6-AH.

Kringles 2 and 4 each contain three tryptophans of which two form the binding pocket for 6-AH. The third tryptophan is close to the other two. Kringle 5 contains two tryptophans. Binding of 6-AH to kringle 5 is a replacement reaction. 6-AH exchanges for the side chain of lys-50; one negative charge is introduced into the structure. The actual binding site consists of W62 and Y72. W25 is close by, within 3 Å. Of the 19 tryptophan the environments of nine do not change because they are too far away from the 6-AH; the two tryptophans in kringle 5 go from a less polar to a more polar environment when 6-AH is present. The remaining eight tryptophans go from more to less polar when 6-AH binds. Referring back to row one of Table 1, the hydrophobic shift on adding 6-AH can be explained on the basis of the eight plus two tryptophans whose environments change.

The tyrosine data are more difficult to rationalize. There are 34 tyrosines in DPGN; ten are in the proteolytic domain and three are in the NTP.

As closed DPGN goes from low to higher temperature the protein assumes a more compact structure but the absorption data indicate that the average tyrosine does not sense a change when this occurs (Figure 3).

The major surprise is in the response of the protein vis a vis the closed/open transition. The absorption data from the closed DPGN indicate that the average environment of the tyrosines is close to that of water. This shifts to 25%–33% methanol on addition of 6-AH. The 10 tyrosines in the proteolytic domains are not likely the cause of this change, nor are the tyrosines in the NTP, nor are the tyrosines in kringle 3 which does not bind 6-AH.

Kringle 1 has five tyrosines, kringle 2 has three, kringle 4 has four and kringle 5 has five. The proximity of the tyrosines to the tryptophans is similar to the pattern for kringle 1 shown in Figure 6. Kringles 1,2 and 4 exchange water for 6-AH on binding the latter; kringle 5 exchanges the R-group of lysine 50 for 6-AH on binding. The binding kringles 1,2 and 4 become less polar in the regions of the binding pockets whereas kringle 5 probably becomes somewhat more polar. The last complication is a result of the tyrosine in the kringle3/kringle4 linker region. This certainly changes environment when 6-AH binds to the kringles but it is not clear how the linker region environment changes as it is not directly involved in binding 6-AH. What is clear is that the environments and the spectra of the aromatic residues of DPGN change subtly as the macromolecule is subjected to changing temperature and ligation and that the spectral changes can be detected and identified using 4th derivative UV absorption spectroscopy.

## Figures and Tables

**Table 1.** The comparative methanol concentration sensed by the tryptophan and tyrosine residues of DPGN when subjected to temperature change or when the closed molecule is forced to open.

**Relaxed**	**Closed**	**Open**	**Closed**	**Open**	**Compact**
0 °C Trp	20%	35%	25%	40%	25 °C Trp
0 °C Tyr	0%	25%	3%	33%	25 °C Tyr

## Abbreviations:

6-AH: 6-aminohexanoate, a homolog of lysine
DPGN: dog plasminogen
HPGN: human plasminogen
NTP: the N-terminal peptide of DPGN (It consists of the first 77 amino acids in the protein)
